# A Computational Data Mining Strategy to Identify the Common Genetic Markers of Temporomandibular Joint Disorders and Osteoarthritis

**DOI:** 10.1055/s-0042-1743571

**Published:** 2022-03-09

**Authors:** Vijayashree Priyadharsini Jayaseelan, Paramasivam Arumugam

**Affiliations:** 1 Clinical Genetics Lab, Cellular and Molecular Research Centre, Saveetha Dental College and Hospital, Saveetha Institute of Medical and Technical Sciences, Saveetha University, Poonamallee High Road, Chennai, Tamil Nadu, India; 2 Molecular Biology Lab, Cellular and Molecular Research Centre, Saveetha Dental College and Hospital, Saveetha Institute of Medical and Technical Sciences, Saveetha University, Poonamallee High Road, Chennai, Tamil Nadu, India

**Keywords:** computational approach, osteoarthritis, temporomandibular joint disorders, inflammation, genetic markers

## Abstract

**Statement of Problem** Prosthodontic planning in patients with temporomandibular joint disorders (TMDs) is a challenge for the clinicians.

**Purpose** A differential biomarker identification could aid in developing methods for early detection and confirmation of TMD from other related conditions.

**Materials and Methods** The present study identified candidate genes with possible association with TMDs. The observational study delineates genes from three datasets retrieved from DisGeNET database. The convergence of datasets identifies potential genes related to TMDs with associated complication such as osteoarthritis. Gene ontology analysis was also performed to identify the potential pathways associated with the genes belonging to each of the datasets.

**Results** The preliminary analysis revealed vascular endothelial growth factor A ( *VEGFA* ), interleukin 1 β ( *IL1B* , and estrogen receptor 1 ( *ESR1* ) as the common genes associated with all three phenotypes assessed. The gene ontology analysis revealed functional pathways in which the genes of each dataset were clustered. The chemokine and cytokine signaling pathway, gonadotropin-releasing hormone receptor pathway, cholecystokinin receptors (CCKR) signaling, and tumor growth factor (TGF)-β signaling pathway were the pathways most commonly associated with the phenotypes. The genes *CCL2, IL6* , and *IL1B* were found to be the common genes across temporomandibular joint (TMJ) and TMJ + osteoarthritis (TMJ-OA) datasets.

**Conclusion** Analysis through computational approach has revealed *IL1B* as the crucial candidate gene which could have a strong association with bone disorders. Nevertheless, several immunological pathways have also identified numerous genes showing putative association with TMJ and other related diseases. These genes have to be further validated using experimental approaches to acquire clarity on the mechanisms related to the pathogenesis.

## Introduction

### Clinical Implications

Identification of molecular biomarkers and the pathway associated with the same is an important strategy to design and develop prognostic tools for complex disorders. Assessing markers could be a herculean task when it comes to human disorders due to heterogeneity and complexity of the disorders. Computational analysis could aid in delineating the markers associated with a disease by predicting interactions, providing a clue toward understanding the pathogenesis of the disease.

Temporomandibular disorders (TMDs) include a heterogeneous group of conditions emanating from the masticatory muscles and mandibular joints resulting in dysfunction and pain. The prevalence of TMDs in the general population is recorded as 21 to 73%, with painful TMD exhibiting a greater prevalence of 65.7% over nonpainful forms reported to be 40.8%. The gender-wise assessment of prevalence of TMD has been recorded as high as 72.4% in females than in male (10.6–68.1%) population. The incidence of the disorder was observed in adults over 18 years of age than the younger ones. 1 Although reports did not provide suggestive evidence on the association between TMDs and malocclusion, a few occlusal traits have been implicated in TMDs. The etiology of factors associated with TMDs can be classified as initiating, predisposing, and perpetuating factors which cause onset of TMDs, increase the risk of TMDs, and enhance progression of TMDs by interfering with the healing process. Although the etiology of TMDs is multifactorial with the involvement of physiological, psychosocial, neurological and cognitive factors, heredity, or genetic factors play a very important role in the predisposition of the disorder. Despite the fact that there are numerous reports related to TMDs based on epidemiology, gender, clinical presentations, and others, only a very few records are available to support this hereditary predisposition to TMDs.

To bridge the gaps and address the lacunae that lies in the temporomandibular joint (TMJ) research related to genetic predisposition, the authors have embarked on an exploration employing computational tools to identify the “hub of genes”' which are candidate genes whose deregulation precipitates into TMD phenotypes. Additionally, osteoarthritis (OA) is considered to be one of the major risk factors often related to TMDs. Orthodontists report a higher incidence of TMDs in their patients suffering from OA. 2 OA is a progressive disease commonly found in women who lead to disturbances in health and day-to-day performances due to severe functional decline. A very recent study by Kang demonstrated the immunological pathway related to TMJ + OA (TMJ-OA) in young females. The RNA-sequencing analysis conducted by recruiting 24 females with TMJ-OA and 11 age- and gender-matched control group revealed nearly 41 differentially expressed genes. These genes were further clustered into 16 ontology terms. A hub of six candidate genes, that is, *HLA-C, HLA-F, CXCL8, IL11RA* , *IL13RA1* , and *FCGR3B* were identified. They were shown to be regulating pathways related to inflammation, autoimmunity, and dysregulation of T-cell functions. 3 4 Thus, clinical experimental procedures coupled with in silico analysis pave way in selecting the most appropriate gene clusters associated with the disease phenotype, thereby enabling the development of therapeutic leads targeting these gene products.

The authors hypothesize that TMDs and OA could have common pathways culminating in the degenerative process. Hence a comprehensive assessment of genes related to the phenotypes TMJ, TMJ-OA, and generalized OA (G-OA) was performed for deriving a better understanding of the converging molecular mechanisms underlying these closely and commonly occurring disorders. Further, the putative factors behind increased prevalence of OA and TMJ-related OA have been discussed in terms of genetic predisposition and underlying pathways. The present study is the first of its kind, wherein computational tools were used to assess data to identify those hub of genes and their encoded protein products which could be considered for targeted therapy. The preliminary data have been analyzed in different dimensions to tag high priority genes associated with susceptibility to TMJs.

## Methods

### Collection of Data

DisGeNET is a user friendly platform which is a collection of publicly available information on genes and variants associated with human diseases. 5 6 7 It is an integrated domain with data curated from the Genome Wide Association Studies (GWAS), animal models, and text-mining approaches. Also, additional metrics are provided to enable prioritization of genotype and phenotype associations. The present version (v7.0) comprises 1,134,942 gene-disease associations (GDAs) which include 21,671 genes encompassing 30,170 diseases or traits and 3,69,554 variant associations (VDAs).

### Cytogenetic Location of Genes

GeneCards is an integrative platform which provides information on all predicted genes of the human genome. The database integrates the information from approximately 150 web sources, hosting genomic, transcriptomic, proteomic, genetic functional, and clinical information about the genes. The frequency of genes in each of the dataset across the genome was presented as a graph to denote the involvement of a specific chromosome with the type of disorder. 8

### Protein Interaction Analysis

The STRING (v11.0) database hosts a range of known and predicted protein–protein interactions. The interactions may be of two main types: direct (physical) and indirect (functional) associations. The information gathered in the database is derived from predictions performed using computational tools, text mining, and data mining from other primary databases. 9

### Gene Ontology Analysis

Gene ontology analysis was performed by using the PANTHER database (v16.0; Protein ANalysis THrough Evolutionary Relationships). User-defined query lists of genes from each of the datasets were fed as a batch to identify the functional classification of the genes. Classification based on pathway was conducted to identify the potential pathways associated with the genes. 10 11

## Results

### DisGeNET Analysis

**Fig. 1:**
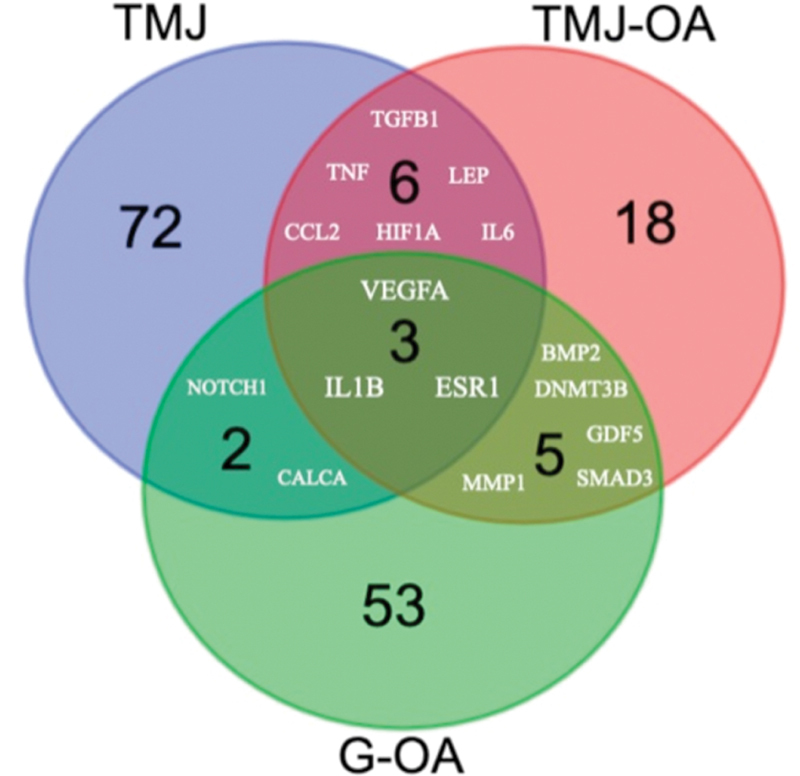
The Venn diagram showing common genes associated with both TMJ, G-OA, and TMJ-OA. G-OA, generalized-osteoarthritis; TMJ, temporomandibular joint; TMJ-OA, temporomandibular joint-osteoarthritis.

The three different datasets unique for each phenotype, that is, (1) TMJ (C0039494), (2) TMJ-OA (C4552513), and (3) G-OA (C1384584) datasets with 83, 32, and 63 genes, respectively, were selected for the present analysis. The catechol-O-methyltransferase (COMT; TMJ), interleukin 1 β (IL1B; TMJ-OA), and collagen type-II α 1 chain (COL2A1; G-OA) were found to exhibit high-gene-disease-association score for the corresponding dataset analyzed. The preliminary analysis revealed common genes associated with all three datasets were vascular endothelial growth factor A ( *VEGFA* ), *IL1B* , and estrogen receptor 1 ( *ESR1* ; Table 1 ; Fig. 1 ). Similarly, unique collections of genes specific for each of the phenotypes were also identified ( Table 1 ).

**Table 1:** The number of genes implicated in each of the category studied and common genes identified among different combinations of datasets

Names	Total number of genes	Genes
G-OA, TMJ, TMJ-OA	3	*VEGFA, IL1B, ESR1*
G-OA, TMJ	2	*NOTCH1, CALCA*
TMJ, TMJ-OA	6	*TNF, TGFB1, LEP, CCL2, HIF1A, IL6*
G-OA, TMJ-OA	5	*DNMT3B, MMP1, SMAD3, GDF5, BMP2*
TMJ	72	*EREG, SHOX2, MTOR, BECN1, GRHL3, HTR2A, DCTN4, TXNRD2, PITX2, ESRRB, TNFRSF11B, CCL5 MAP1LC3A, KHDRBS1, LTA, ADRB2, CMYA5, TTN, CAPN3, SLC6A4, GLI3, MRAS, AMY1C, TAL1, DRD2, NGF, CT, AMY1A, TRIP12, ELN, TIMP1, CTRL, CALCR, GTF2H1, NUP62, MAP1LC3B, ENPP1, ESR2, VCP, SCPEP1, BAHCC1, GSTM1, ANKK1, DMD, LTBP3, DRD4, CRP, RARRES2, SQSTM1, GSTT1, ANKH, MTHFR, COL4A3, PTH, TNFRSF11A, ITGAL, CXCL8, EGR1, COMT, MAP4K3, HPGDS, EGFR, NFKB1, DKK1 IL1RN, BTF3P11, CCDC88A, KRT7, SCLY, MMP9, AMY1B, IL11*
G-OA	53	*VDR, BMP4, POLDIP2, HCK, SFRP4, RNF19A, GNL3, GRAP2, MEFV, CCL8, DNASE1L3, TNFAIP6, HFE BID, COL9A1, PART1, IGF1, MIR146A, MAPK14, ROM1, PHB, CLU, PITX1, CNGB1, COL2A1, PTH2R, MMP13, TPSG1, HLA-DRB1, IL1A, LRRC32, MMP3, SERPINA3 PTK2B, HLA-B, CRK, MICAL3, NAMPT, MAPKAPK2, HLA-A, AIMP2, AHSA1, BMPR1A, MAPK1, TLR10, SPP1, DNMT1, SERPINA1, GHR, CTNNB1, GH1, NODAL, MGP*
TMJ-OA	18	*IL17A, NPAS2, SSRP1, DMP1, PER2, SETD2, MIR140, CHST11, HOTAIR, MAPK3, HES5, CCR5, CCL20, IL22, TNFSF11, ESRRG, CD68, RUNX2*

Abbreviations: G-OA, generalized-osteoarthritis; TMJ, temporomandibular joint; TMJ-OA, temporomandibular joint-osteoarthritis.

### Frequency Distribution of Genes on the Chromosomes

**Fig. 2:**
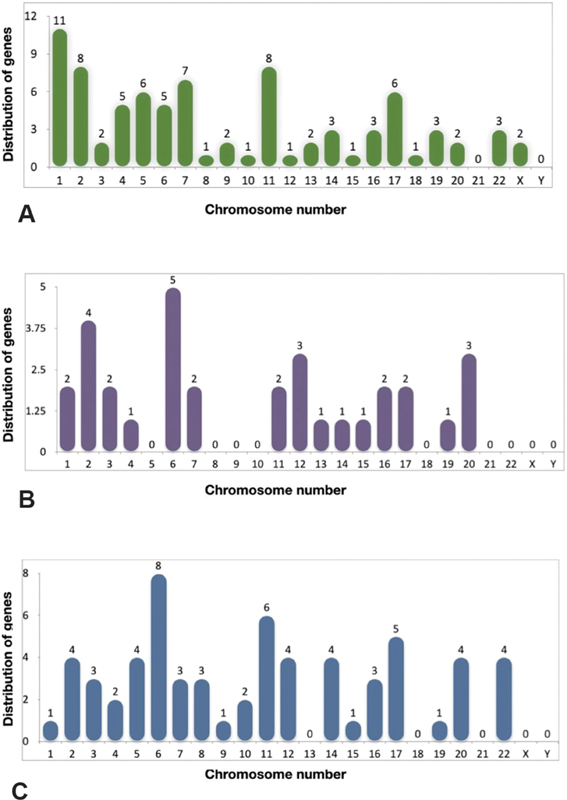
( **A** ) The distribution of genes across the genome in the TMJ dataset (C0039494), ( **B** ) TMJ-OA (C4552513), and ( **C** ) G-OA (C1384584). G-OA, generalized-osteoarthritis; TMJ, temporomandibular joint; TMJ-OA, temporomandibular joint-osteoarthritis.

The frequency distribution of genes from different datasets was identified using GeneCards and clustered based on their location on each of the 23 chromosomes ( Figs. 2A–C ). Eleven genes on chromosome 1 (TMJ), 5 genes on chromosome 6 (TMJ-OA), and 8 genes on chromosome 6 (G-OA) were found to be present in the respective datasets. In line with the observation made, chromosome 6 was found to harbor more genes associated with generalized OA and TMJ. Cumulative effect of variations in these genes might increase the susceptibility to TMDs.

### Protein Interaction Analysis

**Fig. 3:**
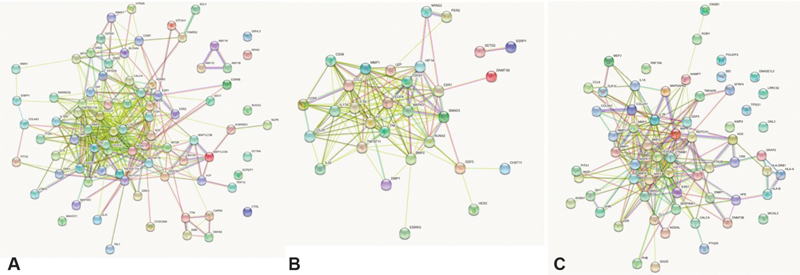
( **A** ) The protein network interaction of genes in the TMJ dataset (C0039494), ( **B** ) TMJ-OA (C4552513), and ( **C** ) G-OA (C1384584). G-OA, generalized-osteoarthritis; TMJ, temporomandibular joint; TMJ-OA, temporomandibular joint-osteoarthritis.

The analysis using STRING database returned protein interaction network for each of the dataset investigated. The TMJ dataset had two gene clusters with the majority of genes interacting in one cluster and amylase α 1A ( *AMY1A* ), 1B ( *AMY1B* ), and 1C ( *AMY1C* ) forming a second cluster interacting with each other. Seven other genes, such as *GRHL3, MRAS, SHOX2, DCTN4, SCPEP1, CTRL* , and *BAHCC1* , were found to remain as independent entities without showing any interactions with either of the clusters ( Fig. 3A ). Similarly TMJ-OA had two gene clusters with *SETD2* (SET domain-containing 2, histone lysine methyltransferas) and *SSRP1* (structure-specific recognition protein 1), forming a secondary cluster ( Fig. 3B ). The G-OA dataset showed a single cluster with majority of genes and a few genes which were found to act independently, that is, *POLDIP2, BID, DNASE1L3, LRRC32, TPSG1, GNL3, AIMP2, MICAL3* , and *RNF19A* ( Fig. 3C ).

### Gene Ontology Analysis

**Fig. 4:**
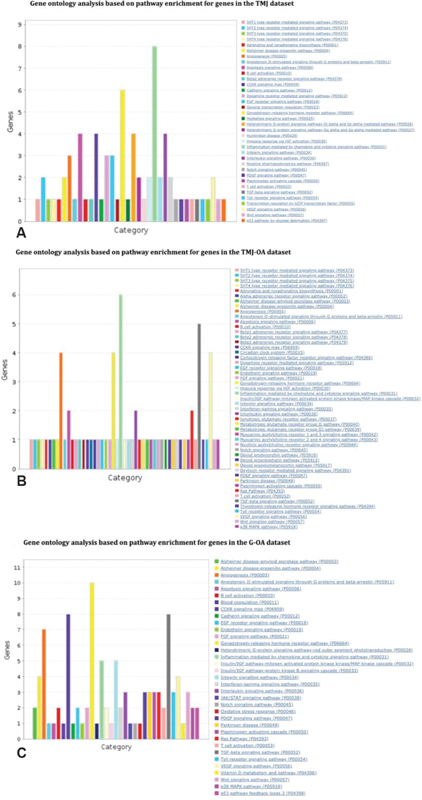
The gene ontology results depicting the cluster of genes contained in each of the pathway in the ( **A** ) TMJ (C0039494), ( **B** ) TMJ-OA (C4552513), and ( **C** ) G-OA (C1384584) datasets. G-OA, generalized-osteoarthritis; TMJ, temporomandibular joint; TMJ-OA, temporomandibular joint-osteoarthritis.

The gene ontology analysis identified 36 pathways in the TMJ set of genes with the highest cluster being found in the inflammation mediated by chemokine and cytokine signaling pathway with eight genes, that is, *CCL2, ITGAL, CXCL8, CCL5, NFKB1, LTA, IL6* , and *IL1B* . The second highest cluster was that of gonadotropin-releasing hormone receptor pathway, comprising six genes, that is, *MAP4K3, EGFR, EGR1, PITX2, TGFB1* , and *DRD2.* Similarly, on analysis of TMJ-OA dataset, a total of 51 pathways were revealed of which the highest cluster was observed as possessing six genes of the inflammation mediated by chemokine and cytokine signaling pathway, *CCR5, CCL2, CCL20, MAPK3, IL6* , and *IL1B* , and the second highest being tumor growth factor (TGF)-β signaling pathway with five genes, that is, *BMP2, SMAD3, GDF5, TGFB1* , and *MAPK3.* Subsequently, the observation derived from G-OA dataset revealed 35 pathways with the highest cluster with 10 genes ( *BMP2, BMP4, SMAD3, BMPR1A, PITX1, CTNNB1, MAPK1, IGF1, MAPK14,* and *PTK2B* ) belonging to gonadotropin-releasing hormone receptor pathway. The second highest cluster included two pathways, the cholecystokinin receptors (CCKR) signaling ( *MMP3, CLU, CRK, CTNNB1, CALCA, MAPK1, MAPK14,* and *PTK2B* ) and TGF-β signaling pathway ( *BMP2, BMP4, SMAD3, GDF5, BMPR1A, MAPK1, MAPK14,* and *NODAL* ) with eight genes each ( Fig. 4A–C ).

## Discussion

The etiology of TMD is multifactorial. Several factors, such as trauma, osteoarthritis, neuromuscular, mechanical displacement, psychophysiological, psychosocial, and hereditary components, have been identified as causes of TMD. 1 Among the predisposing, initiating, and perpetuating factors, a predisposing factor with a special emphasis on the genetic component influencing the susceptibility of TMD has been considered in the present study. Computational analysis is a growing field where even a small piece of information can contribute immensely to the research intended to alleviate the discomforts experienced by patients suffering from TMDs. The present study is one such attempt conducted to identify high priority genes in close association with TMD. The DisGeNET analysis showed *COMT* (TMJ), *IL1B* (TMJ-OA), and *COL2A1* (G-OA) to exhibit high gene-disease association scores in each of the dataset investigated. Meta-analysis conducted by Brancher and colleagues identified *COMT* to be significantly associated with TMD. They reported two polymorphisms *rs6269* and *rs9332377* to be related to myofascial pain or myofascial pain with painful TMD respectively. 12 COMT is an enzyme which is involved in the catalysis of catecholamines, such as dopamine, epinephrine, and norepinephrine. The forms of COMTs, that is, soluble COMT and membrane bound COMT, are encoded by the gene located at the cytogenetic loci 22q11.21. Numerous polymorphisms in the coding, intergenic, and promoter regions of the COMT gene have been found to be associated with stress and painful conditions. The distribution of genes across different datasets revealed chromosomes 1 and 6 to harbor the highest frequency of genes associated with the disease phenotypes. The high scoring gene in TMJ-OA group was *IL1B* which encodes cytokines that participate in immunoregulation and inflammatory processes. The *COL2A1* gene encoding type-II collagen is located at 12q13.1-q13.2. 13 Although once considered to be encoding for structural component, it was recently established that COL2A1 can act as an extracellular signaling molecule capable of suppressing chondrocyte hypertrophy. 14

The intersection of 3 datasets ( *http://bioinformatics.psb.ugent.be/cgi-bin/liste/Venn/calculate_venn.htpl* ) revealed common genes associated with all three datasets which were *VEGFA* , *IL1B* , and *ESR1* ( Fig. 1 ). The formation of small blood vesicles within the joint cavity is most frequently observed in case of synovitis. A recent study by Wang and colleagues provided evidence about the involvement of VEGF and FGF-2 in inducing angiogenesis in joints. The study findings also suggested that administration of intra-articular dose of hyaluronic acid (HA) might alleviate synovotis by targeting VEGF, whose level was found to be high before treatment and reduced significantly after treatment with HA. 15

Cytogenetic location of the genes of TMJ dataset, that is, *CRP, AMY1A, AMY1B, AMY1C, GSTM1, GRHL3, MTOR, MTHFR, NGF, TAL1* , and *KHDRBS1* were found on chromosome 1 ( Fig. 2A ). On the other hand, *IL17A, RUNX2* , and *TNF* were all clustered on chromosome 6 in TMJ-OA ( Fig. 2B ). The genes *COL9A1, HFE, HLA-A, HLA-B, DRB1* , and *MAPK14* were found to be on chromosome 6 ( Fig. 2C ). Interestingly, *ESR1* and *VEGFA* were found to be the overlapping genes in TMJ-OA and G-OA sets. Although the heritability data for TMJ is not well established in different populations, a consolidated data on the involvement of chromosomes and genes have been addressed in the present study. As with TMJ and associated disorders being multifactorial in nature, cumulative effects of all the variants occurring in the genes mentioned above can contribute toward the magnitude of the disorder as TMJ with mild-to-moderate symptoms or more severe forms of TMJ eventually leading to serious disabilities.

Finally, the gene ontology analysis revealed one pathway which was common for TMJ and TMJ-OA dataset and one pathway common to TMJ-OA and G-OA which was the TGF-β signaling pathway. Gonadotropin-releasing hormone receptor pathway was found to be common between TMJ and G-OA. The other pathway of significant importance was the CCKR signaling pathway. The protein interaction network analysis provided information on gene clusters in two datasets, TMJ and TMJ-OA, whereas the G-OA showed a single cluster with the highest number of independent genes alongside the cluster as depicted in Fig. 3C . Gender predilection is a common observation in OA, where females show a greater prevalence than males. The possible reason for greater prevalence can be attributed to hormone estrogens. Estrogens are known to regulate several biological processes including reproduction, differentiation, development, and cell growth. 16 Cell signaling mediated by estrogens requires two types of receptors, (1) ERa and (2) ERb, encoded by *ESR1* and *ESR2* genes. 17 The ERa is expressed in mandibular condylar cartilage which describes their possible role in the development of TMDs. 18 19 A very recent study by Dalewski demonstrated the association of *rs1643821 ESR1* gene polymorphism and *rs1800629 TNF-α* gene polymorphism with disc displacement in TMD. The study recruited 124 Caucasian patients with TMDs and 126 control patients free of TMDs. Among the two polymorphisms, *rs1643821 (ESR1)* exhibited a significant association conferring susceptibility to anterior disc displacement observed in TMDs. 20 Pinto Fiamengui and team assessed the role of inflammatory and pain-related gene polymorphisms in Brazilian patients who presented with TMDs. About 131 TMDs patients and 1347 normal patients were included in the cross-sectional study. Allelic discrimination among variants of different genes *COMT* ( *rs4680* ), *IL-1β* ( *rs1143634* ), *IL6* ( *rs1800795* ), and *TNFA* ( *rs1800629* ) were assessed. Of the four Single nucleotide polymorphisms (SNPs), TNF- α and IL6 showed a significant association with TMD and sensitivity to pain, respectively. 21

About 1.2, 6.25, and 14.28% of the total genes in TMJ, TMJ-OA, and G-OA datasets, respectively, were found to act independently and were not involved in the interactions. Genes of the G-OA lying out of the cluster could make the phenotype more diverse involving pathways which are yet to be explored. As reported by researchers worldwide, inflammatory cytokine and chemokine pathways serve as a converging point between TMJ and TMJ-OA phenotypes. As anticipated, the gonadotropin-releasing pathway was common between TMJ and G-OA which could explain the risk of TMJ and arthritis in females rather than in male population. Induction of inflammatory pathways is a common observation in TMDs. Chronic inflammatory process driven by chemokines and cytokines are the well-known factors implicated in the resorption of bones and damage to adjoining tissues. Among all the cytokines involved, IL1B has been found to show a greater frequency of occurrence in each of the analyses made. Almeida and colleagues determined the association of IL1B expression with TMJ employing immunohistochemical analysis. A total of 39 samples including eight control and 31 obtained from patients with anterior disc displacement with and without reduction. A statistically significant association was observed between the two groups implying the fact that IL1B possesses a major role as a precipitating factor in TMJ. 22

Amidst all the observations made in the present study, the authors demonstrate an interesting network of genes involved in the CCKR signaling pathway. The pathway included *MMP3, CLU, CRK, CTNNB1, CALCA, MAPK1, MAPK14* , and *PTK2B* . When most of the candidate genes were thought to be involved in physiological, immunological, and metabolic functions, the CCKR pathway has been involved in a psychological process that is mostly implicated as one of the risk factors in TMDs. Stress, fear, and anxiety are known to be controlled by CCK. The CCK1 receptor binds to sulphated CCK peptide hormone, widely distributed in the gastrointestinal tract and brain of mammals. 23 A recent review compiled by Florjański and Orzeszek provided substantial evidence supporting the fact that psychological derangements play an inherent role in the development and progression of TMDs. 24 Earlier studies on the CCK showed that it inhibits gonadotropin releasing cell's neuronal activity through CCKRs. Thus, it is clear that a trio of psychological, hormonal, and genetic factors makes a female more vulnerable to G-OA phenotype. 25

Despite the fact that the present study is designed based on data generated from experimental procedures, datamining, and text mining, there could be genes or pathways which have not been explored to a greater extent. Second, since the results are not based on a specific population or ethnic group, the observation made on potential candidate genes can differ across geographical locations, as other extraneous factors, such as gender, habits, dwelling place, income, level of education, food, life style, and others, might act as potential modifiers of the hereditary factors. Third, information on more intricate mechanisms, such as exosomes, 26 27 28 RNA modifiers, epigenetic, and epitranscriptome marks 29 30 implicated in other inflammatory diseases are not dealt with in a comprehensive manner. Further, the phenotype TMD includes a heterogeneous group of symptoms. TMD's classifications are intra-articular, extra-articular, musculoskeletal, and psychological problems, and the etiology of each classification is different; hence, more specific experimental approach should be designed to identify a panel of genes to be analyzed in a specific type to recognize vital genetic markers contributing to susceptibility of such disorders.

## Conclusion

Genetic basis of TMDs still remains to be a debatable topic. Taken together, the present study reveals a hub of genes based on cluster and pathway analysis to be associated with TMDs. These potential genes include, *COMT, IL1B, COL2A1, VEGFA* , and *ESR1.* Additionally, genes related to inflammatory pathways do have a vital role to play in the development of the disorder. Among all the data analyzed, it is of interest to know that *IL1B* can be regarded as one of the major precipitating factors for TMJ and OA. Having addressed all the pros and cons of the present study, the authors suggest data generated through experimental evidence to prove the association *IL1B* with this disease phenotype. Advancements in next generation sequencing analysis, whole genome and exome sequencing, RNA sequencing, transcriptomics, and epitranscriptomics studies are sure to provide evidence-based reports on the heritability index of TMJs and related disorders.
